# Bit-Level Construction for Multiplicative-Repetition-Based Non-Binary Polar Codes †

**DOI:** 10.3390/e27040377

**Published:** 2025-04-02

**Authors:** Rongchi Xu, Peiyao Chen, Ling Liu, Min Zhu, Baoming Bai

**Affiliations:** 1 The State Key Laboratory of ISN, Xidian University, Xi’an 710071, China; rcxu@stu.xidian.edu.cn (R.X.); peiyaochen24@gmail.com (P.C.); mzhu@xidian.edu.cn (M.Z.); 2 The Guangzhou Institute of Technology, Xidian University, Guangzhou 510555, China; liuling@xidian.edu.cn

**Keywords:** non-binary polar codes, channel degradation, construction algorithms, probabilistic shaping

## Abstract

In this paper, we discuss non-binary polar codes using a 2×2 matrix over a Galois field GF($2^{q}$) as the kernel. Conventional construction of non-binary polar codes divides the synthesized channels into frozen channels and information channels. Each information channel carries one symbol, i.e., *q* bits. However, there are many middle channels with insufficient polarization, which cannot carry one symbol of *q* bits but only *i* bits, 1≤i<q,i∈Z, at finite block length. In this paper, we consider bit-level construction for multiplicative repetition (MR)-based non-binary polar codes and propose a bit-level construction based on the two following methods. We first calculate the error probability and channel capacity lower bound of each synthesized channel based on the channel degradation method, and then determine both the number and index of the carried bits for each synthesized channel according to the symbol error probability and capacity. To reduce complexity, we also introduce a Monte-Carlo method. We compute the error probability of each synthesized channel carrying *i* information bits and select the optimal construction that can minimize the union bound of the error probability. Finally, an improved construction-based probabilistic shaping method for MR-based non-binary polar codes is considered. Simulation results show that the proposed construction significantly improved the decoding performance compared with the conventional construction scheme.

## 1. Introduction

Binary polar codes, proposed by Arıkan, can achieve symmetric capacity of binary-input discrete memoryless channels (BI-DMCs) [1]. However, the performance of polar codes at finite block lengths is unsatisfactory with the successive cancellation (SC) decoding algorithm. In order to improve the performance of polar codes in the finite length regime, the cyclic redundancy check (CRC)-aided successive cancellation list (CA-SCL) decoding algorithm was proposed in [2,3]. In practice, the CRC-polar concatenation scheme has shown excellent performance at short to medium code lengths with CA-SCL decoding compared with low-density-parity-check (LDPC) codes and Turbo codes. Due to their superior performance, CRC-polar codes have been selected as the coding standard for the control channel in the fifth generation (5G) wireless communication system [4].

It was proved in [5,6] that non-binary polar codes with arbitrary finite input-alphabet sizes can achieve symmetric capacity. It was also shown that all discrete memoryless channels (DMCs) can be polarized by randomized constructions [5]. Subsequently, Mori and Tanaka [7] proposed non-binary polar codes based on an l×l matrix of Reed–Solomon (RS) codes over GF(*p*), where *p* is the power of a prime. They also proved that the exponent of their non-binary polar codes is log(l!)/(llog(l)) for all l≤p, and this exponent can become arbitrarily close to 1 as *l* increases. In 2016, Cheng et al. [8] introduced four-dimensional polar codes based on the RS kernel of GF(4) (RS4). Simulation results demonstrated that non-binary polar codes based on the RS kernel outperformed their binary counterparts. Moreover, binary and RS4-based non-binary kernels were mixed in [9] to reduce the decoding complexity of RS4-based polar codes. However, decoder implementations are not easily generalizable across different field sizes. To overcome this limitation, a novel multiplicative repetition (MR)-based non-binary code was proposed in [10,11,12,13,14]. The conventional decoding algorithm for binary polar codes can be easily extended to this class of non-binary polar codes. In [12], Chen and Bai introduced the concept of two-stage polarization to construct non-binary polar codes, which exhibited comparable performance with RS4-based non-binary polar codes. In [13], Yuan and Steine proposed a kernel selection method to maximize the polarization effect, where the kernel had a fixed multiplicative factor.

For binary polar codes over a binary-input additive white Gaussian noise (BI-AWGN) channel, several construction algorithms exist, including the Monte-Carlo (MC) algorithm [1], Gaussian approximation (GA) algorithm [15], polarized weight (PW) algorithm [16], and others [17,18]. For MR-based non-binary polar codes, the Monte-Carlo construction and channel degradation (CD) construction [19] are mainly used. In 2022, M. Chiu [20] proposed a new construction algorithm for non-binary I-polar codes based on the polarization effect and the block error rate (BLER) upper bound.

To achieve high spectral efficiency, non-binary polar codes combined with multilevel coding (MLC) and bit-interleaved coded modulation (BICM) were proposed in [11,20,21,22]. However, uniformly distributed symbols were considered in these schemes, resulting in a shaping loss up to 1.53 dB in AWGN channels [23,24]. A classical scheme to mitigate this loss is combining probabilistic shaping technique with high-order modulation [25,26]. In 2023, a method for constructing RS-kernel-based non-binary polar codes with shaping was proposed in [27].

This work aimed to improve the performance of MR-based non-binary polar codes at finite block lengths. For  polar codes over GF($2^{q}$), the capacity of many synthesized channels ranges from 0 to *q* bits per channel used at finite block lengths. A single symbol in a synthesized channel may not carry *q* bits, but can carry *i* bits, where 1≤i<q. Inspired by this, we consider bit-level construction for MR-based non-binary polar codes and propose an improved construction based on the CD method and MC method.

The main contributions of this paper are summarized as follows:

First, we revisit CD construction for non-binary polar codes. We then calculate the lower bound of the channel capacity and the symbol error probability for each synthesized channel, which are then used to determine the number of bits each channel can carry. Additionally, we compute the error probability for all transmission patterns and select the optimal pattern that minimizes the error probability of the synthesized channels. Compared with the conventional symbol-level construction, the proposed bit-level construction achieves better BLER performance with the same complexity.

Second, due to the high complexity of CD construction, we also explore the MC method to reduce construction complexity. We compute the error probability of each synthesized channel carrying *i* information bits, 1≤i≤q. Based on this, we select the optimal information channels (i=q) and partial information channels (i<q) to minimize the error probability of the non-binary polar code. Recently, we noticed that the construction in the appendix of [14] is a special case of our bit-level construction-based MC method. Simulation results demonstrated that the proposed construction method improved the BLER performance compared with the original construction and the scheme in [12].

Third, for high spectral efficiency,  MR-based non-binary polar codes combined with BICM and a hybrid MLC and BICM framework [28] are also considered. To improve BLER performance, we integrate probabilistic shaping with BICM and introduce a bit-level construction for shaping MR-based non-binary polar codes.

The article significantly extends the work presented in preliminary form in [29], where we demonstrated the potential of bit-level construction for non-binary polar codes. We provide a more detailed description of bit-level CD and MC constructions. Furthermore, we propose a novel framework combining non-binary polar codes with hybrid MLC and BICM coded modulation, along with a bit-level construction for shaping MR-based non-binary polar codes.

The rest of this paper is organized as follows: Section 2 introduces the notations, definitions, and preliminaries of non-binary polar codes. Section 3 presents the improved construction scheme. In Section 4, the probabilistic shaping method based on the improved construction for multiplicative repetition non-binary polar codes is described. Section 5 provides simulation results for our scheme. Finally, the conclusions are given in Section 6.

## 2. Preliminaries for Non-Binary Polar Codes

### 2.1. Notation and Conventions

Throughout this paper, matrices and vectors are represented by upper-case and lower-case letters in bold, respectively. An *N*-tuple u is denoted as $u=[u_{1},u_{2},...,u_{N}]$. The notation $u_{a}^{b}$ means the sub-vector $\left[u_{a},u_{a+1},...,u_{b}\right]$ if b≥a and a null vector otherwise. The calligraphic letter A is used to denote sets, and the complement of set A is $A^{c}$. The cardinality of A is denoted as |A|. In this paper, we consider the Galois fields of characteristic 2, i.e., GF($2^{q}$), where *q* is a positive integer.

### 2.2. Binary Polar Codes

Given a BI-DMC W:X→Y with input alphabet X and output alphabet Y, where X∈{0,1}, the channel transition probabilities can be defined as W(y|x),x∈X,y∈Y. If *W* is a symmetric channel, the symmetric capacity and the Bhattacharyya parameter of the B-DMC *W* can be defined as

(1) $$C\left(W\right)≜\sum_{y\in Y}\sum_{x\in X}\frac{1}{2}W\left(y|x\right)\log\frac{W(y|x)}{\frac{1}{2}W\left(y|0\right)+\frac{1}{2}W\left(y|1\right)}$$

and

(2) $$Z\left(W\right)≜\sum_{y\in Y}\sqrt{W(y|0)W(y|1)},$$

respectively.

Assume that the code length is $N=2^{n}$, the information sequence length is *K*. With the channel polarization, the *K* most reliable synthesized channels $W_{N}^{\left(i\right)},i\in A$ are used to transmit information bits, and the others are used to transmit the frozen bits, where |A|=K. The encoding of a polar code can be described by

(3) $$x_{1}^{N}=u_{1}^{N}F_{N}=u_{1}^{N}F_{2}^{⊗n}B_{N},$$

where the input sequence $u_{1}^{N}$ contains *K* information bits and N−K frozen bits, the matrix $F_{N}$ is the N×N generator matrix, $B_{N}$ is a permutation matrix [1], ⊗ denotes the Kronecker operation, and the Arıkan kernel $F_{2}$ is given by

(4) $$F_{2}=\left[\begin{array}{cc} 1 & 0\\ 1 & 1 \end{array}\right].$$

The channel splitting operation splits the vector channel $W^{N}$ into *N* polarized channels $W_{N}^{\left(j\right)}:X\to Y\times X^{j-1},1\leq j\leq N$. The transition probability of the *j*-th polarized channel $W_{N}^{\left(j\right)}$ is defined as

(5) $$W_{N}^{\left(j\right)}\left(y_{1}^{N},u_{1}^{j-1}|u_{j}\right)≜\sum_{u_{j+1}^{N}\in X^{N-j}}\frac{1}{2^{N-1}}W_{N}\left(y_{1}^{N}|u_{1}^{N}\right).$$

### 2.3. MR-Based Non-Binary Polar Codes

We extend binary polar codes to non-binary polar codes by considering the following 2×2 kernel $G_{2}$ over GF($2^{q}$), which is given by

(6) $$G_{2}=\left[\begin{array}{cc} 1 & 0\\ \alpha^{t} & 1 \end{array}\right],$$

where α is the primitive element of GF($2^{q}$) and $0\leq t<2^{q}-1$. Note that, in the case of $\alpha^{t}=1$, $G_{2}$ has the same form as the Arıkan kernel $F_{2}$. In the case of $\alpha^{t}\neq 1$, we call $G_{2}$ an MR-based matrix for convenience. In this paper, the generator matrix with code length $N=2^{n}$ symbols is given by

(7) $$G_{N}=G_{2}^{⊗n}=\left[\begin{array}{cc} G_{2}^{⊗(n-1)} & 0\\ \alpha_{n}G_{2}^{⊗(n-1)} & G_{2}^{⊗(n-1)} \end{array}\right],n\geq 2,$$

where $\alpha_{n}=\alpha^{2^{n-1}}$ and t=1 in the matrix $G_{2}$.

**Figure 1:**
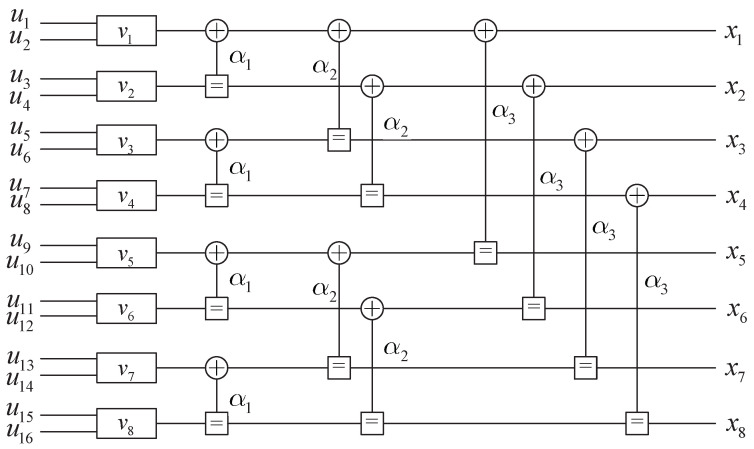
Factor graph of the MR-based polar codes over GF(4).

The encoding of non-binary polar codes can be described as

(8) $$x_{1}^{N}=v_{1}^{N}G_{N}=v_{1}^{N}G_{2}^{⊗n}B_{N},$$

where $v_{1}^{N}$ denotes the *N* input symbols of GF($2^{q}$). Let $u_{1}^{N_{b}}=u_{1}^{N\times q}$ represent the binary representation of $v_{1}^{N}$. The factor graph of the $G_{8}$ MR-based polar codes over GF(4) is shown in Figure 1.

(9) $$\begin{array}{cc} W_{l}^{\left(2j-1\right)}\;\left(y_{1}^{2^{l}},v_{1}^{2j-2}\;|\;v_{2j-1}\right)\;= & \sum_{v_{2j}}\frac{1}{2^{q}}\left\{W_{l-1}^{\left(j\right)}\left(y_{1}^{2^{l-1}},v_{1,\;\mathrm{odd}\;}^{2j-2}+\alpha_{h}v_{1,\;\mathrm{even}\;}^{2j-2}|v_{2j-1}+\alpha_{h}v_{2j}\right)\right.\\ \; & \left.W_{l-1}^{\left(j\right)}\left(y_{2^{l-1}+1}^{2^{l}},v_{1,\;\mathrm{even}\;}^{2j-2}|\;v_{2j}\right)\right\}, \end{array}$$

(10) $$\begin{array}{cc} W_{l}^{\left(2j\right)}\left(y_{1}^{2^{l}},v_{1}^{2j-1}|v_{2j}\right)= & \frac{1}{2^{q}}W_{l-1}^{\left(j\right)}\left(y_{1}^{2^{l-1}},v_{1,\;\mathrm{odd}\;}^{2j-2}+\alpha_{h}v_{1,\;\mathrm{even}\;}^{2j-2}|v_{2j-1}+\alpha_{l}v_{2j}\right)\\ \; & W_{l-1}^{\left(j\right)}\left(y_{2^{l-1}+1}^{2^{l}},v_{1,\;\mathrm{even}\;}^{2j-2}|v_{2j}\right), \end{array}$$

Let W(y∣v) represent the channel conditional probability and $v_{1,odd}^{j},v_{1,even}^{j}$ represent the subvector with odd and even indices, respectively. Due to the similar structure to binary polar codes, the recursive formulas of the symbol-level SC decoding algorithm are given by (9) and (10), where $1\leq j\leq 2^{l}$, $\alpha_{h}=\alpha^{2^{h-1}},h=n-l+1,1\leq l\leq n$, and $W_{0}^{\left(1\right)}\left(y|v\right)=W\left(y|v\right)$. Assuming that the transmission probabilities of all symbols are equal, for the conventional symbol-level construction with the information symbol set A and frozen symbol set F, the SC decoder generates its decision ${\hat{v}}_{i}$ by

(11) $${\hat{v}}_{i}=\left\{\begin{array}{cc} \arg\max_{\beta \in \mathrm{GF}(2^{q})}W_{n}^{i}\left(y_{1}^{N},v_{1}^{i-1}|\beta \right), & i\in A\\ \;\mathrm{value}\;\mathrm{of}\;\mathrm{the}\;\mathrm{frozen}\;\mathrm{symbol}\;v_{i}, & i\in F \end{array}\right..$$

## 3. Bit-Level Construction for Non-Binary Polar Codes

In this section, we introduce the bit-level construction for MR-based non-binary polar codes and propose improved bit-level constructions based on the CD method and the MC method, respectively.

### 3.1. Capacity-Based Bit-Level Construction

**Figure 2:**
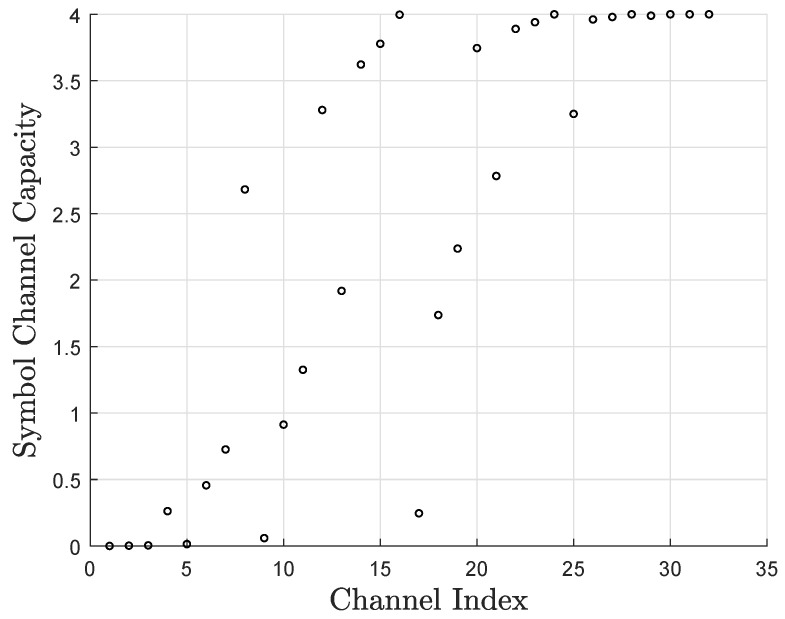
Synthesized channel capacities of non-binary polar codes over the erasure channel.

As for the symbol-level construction of non-binary polar codes over GF($2^{q}$), the *K* most reliable channels are used to carry the information symbols, and each channel carries one symbol of *q* bits. However, at finite block lengths, the capacities of considerably many synthesized channels lie in the range [1,q−1]; i.e., they are not bad enough to be frozen but are not good enough to carry *q* bits per symbol. Through simulations, we found that they are capable of carrying *i*-bit partial symbols for a certain integer 1≤i<q, where *i* is determined by the channel capacity of each synthesized channel, as shown in Figure 2. For the $2^{q}$-ary erasure channel with erasure probability ϵ, the erasure probability of each synthesized channel can be calculated by

(12) $$\begin{array}{cc} \; & \epsilon \left(W_{N}^{\left(2j\right)}\right)=\epsilon {\left(W_{N/2}^{\left(j\right)}\right)}^{2}\\ \; & \epsilon \left(W_{N}^{\left(2j-1\right)}\right)=2\epsilon \left(W_{N/2}^{\left(j\right)}\right)-\epsilon {\left(W_{N/2}^{\left(j\right)}\right)}^{2}, \end{array}$$

with $\epsilon (W_{1}^{1})=\epsilon$. The channel capacity of each synthesized channel is $C\left(W_{N}^{\left(j\right)}\right)=q\left(1-\epsilon \left(W_{N}^{\left(j\right)}\right)\right)$ bits. As an example, Figure 2 shows the synthesized channel capacities of 16-ary polar codes over the erasure channel, where N=32 and the erasure probability ϵ=0.4. For a conventional symbol-level construction, we choose *K* synthesized channels with the largest capacity $C(W_{N}^{\left(j\right)})$ to carry the information symbols. In order to make full use of every synthesized channel, it is reasonable to make the *j*-th channel carry *i* bits if $\lfloor C\left(W_{N}^{\left(j\right)}\right)\rfloor \geq i$. We propose a bit-level construction that considers both performance and complexity. Assuming that I(j) is the number of bits carried over the *j*-th channel, we set

(13) $$I\left(j\right)=\lfloor C\left(W_{N}^{\left(j\right)}\right)\rfloor ,$$

where ⌊C⌋=max{m∈Z|m≤C}. Then, we sort each synthesized channel in descending order based on $C(W_{N}^{\left(j\right)})$ and generate the information symbol set $A_{1}$ for bit-level construction, such that $|A_{1}|\geq K$ and

(14) $$\sum_{j\in A_{1}}I\left(j\right)=Kq\left(bits\right).$$

Note that Equation (14) always holds if Kq information bits can be transmitted correctly at a given channel state.

Although the constructions developed for erasure channels remain functionally valid in AWGN channels, their performance is suboptimal. For enhanced accuracy, we use the CD method [18,19] to obtain the lower bound of the channel capacity of each synthesized channel. Since the kernel matrix $G_{2}$ is different from that in [19], the channel transformations $W^{-}$ and $W^{+}$ can be defined by

(15) $$W^{-}\left(y_{1},y_{2}|v_{1}\right)≜\frac{1}{2^{q}}\sum_{v_{2}=0}^{2^{q}-1}W\left(y_{1}|v_{1}+\alpha_{h}v_{2}\right)W\left(y_{2}|v_{2}\right),$$

(16) $$W^{+}\left(y_{1},y_{2},v_{1}|v_{2}\right)≜\frac{1}{2^{q}}W\left(y_{1}|v_{1}+\alpha_{h}v_{2}\right)W\left(y_{2}|v_{2}\right).$$

Let W:X→Y be a DMC, and let $y_{1},y_{2}$ be two output symbols. Define the channel $Q:X\to Y\setminus \left\{y_{1},y_{2}\right\}\cup \left\{y_{\mathrm{merge}}\right\}$ and the transition probabilities

(17) $$Q\left(y|x\right)=\left\{\begin{array}{cc} W(y|x), & \;\mathrm{if}\;y\in y\setminus \{y_{1},y_{2}\}\\ W\left(y_{1}|x\right)+W\left(y_{2}|x\right), & \;\mathrm{if}\;y=y_{\mathrm{merge}} \end{array}.\right.$$

*Q* is obtained from *W* by replacing $\left\{y_{1},y_{2}\right\}$ with a new symbol $y_{merge}$. Then, Q≼W denote that *Q* is degraded with respect to *W* [18,19]. For MR-based non-binary polar codes, we need to optimize the degraded channel by attaining the smallest rate loss over all Q:

(18) $$\underset{\begin{array}{c} Q:Q≼W\\ \left|\mathrm{output}\left(Q\right)\right|\leq \mu \end{array}}{\inf}C\left(W\right)-C\left(Q\right).$$

Let $j\leq N=2^{n}$ be a nonnegative integer with binary representation ${\langle b_{j,1},b_{j,2},\ldots ,b_{j,n}\rangle }_{2}$. The degradation procedure is described in more detail in Algorithms 1 and 2, where GetOutputwithMinDeltaC(Q) is a search function that finds $y_{1}$ and $y_{2}$ with the smallest capacity difference from *Q*. After that, we obtain the transition probability $Q_{N}^{\left(j\right)}\left(y|v\right)$ of the *j*-th synthesized channel. We define

(19) $$Q_{N}^{\left(j\right)}\left(v|y\right)=\frac{Q_{N}^{\left(j\right)}\left(y|v\right)}{\sum_{v_{0}\in \mathrm{GF}\left(2^{q}\right)}Q_{N}^{\left(j\right)}\left(y|v_{0}\right)},$$

and

(20) $$P_{Q_{N}^{\left(j\right)}}\left(y\right)=\frac{1}{2^{q}}\sum_{v_{0}\in \mathrm{GF}\left(2^{q}\right)}Q_{N}^{\left(j\right)}\left(y|v_{0}\right).$$

Then, we have

(21) $$C\left(Q_{N}^{\left(j\right)}\right)=H\left(V\right)-\sum_{y\in Y}H\left(V|Y=y\right)P_{Q_{N}^{\left(j\right)}}\left(y\right).$$

Since the $C(Q_{N}^{\left(j\right)})$ in (21) is the lower bound of the *j*-th synthesized channel, we set $I\left(j\right)=\lfloor C\left(Q_{N}^{\left(j\right)}\right)+\delta \rfloor$, where δ represents the difference between the capacity and the lower bound, 0≤δ≤0.1. Then, we sort each synthesized channel in descending order based on $C(W_{N}^{\left(j\right)})$ and generate the symbol information set $A_{1}$, $|A_{1}|\geq K$ and $\sum_{j\in A_{1}}I\left(j\right)=Kq\left(bits\right)$.

In addition, we need to determine which bit index carries information when 0<i<q. Let $T_{m}^{\left(j\right)}=1$ represent the information bit carried by the *m*-th index of the *j*-th synthesized channel, and $T^{\left(j\right)}=\left(T_{1}^{\left(j\right)},T_{2}^{\left(j\right)},\ldots ,T_{q}^{\left(j\right)}\right)$ represents the pattern of *j*-th synthesized channel carrying *i* information bits, where $wt(T^{\left(j\right)})=i$ represents the number of ones in $T^{\left(j\right)}$. According to the conditional probability $Q_{N}^{\left(j\right)}\left(y|v\right)$ of the *j*-th synthesized channel, the error probability under the maximum likelihood decoding of pattern $T^{\left(j\right)}$ can be calculated by

(22) $$P_{T^{\left(j\right)}}=\frac{1}{2^{i}}\sum_{y\in Y}\sum_{\begin{array}{c} v^{\prime }\\ v^{\prime }\neq v_{\max}^{\prime } \end{array}}Q_{N}^{\left(j\right)}\left(y|v^{\prime }\right),$$

where

(23) $$v_{\max}^{\prime }=\underset{v^{\prime }}{\arg\max}\;Q_{N}^{\left(j\right)}\left(y|v^{\prime }\right),$$

denotes the decoding result. The pattern $T^{\left(j\right)}$ with the minimum error probability will be selected, and if $T_{m}^{\left(j\right)}=1$, the ((j−1)×q+m)-th bit is an information bit. The details are shown in Algorithms 3 and 4, where ${\langle b_{k,m}\rangle }_{2}$ is the binary representation of a nonnegative integer *k*, and $P_{\max}$ represents the probability that $Q_{N}^{\left(j\right)}\left(y|v_{\max}^{\prime }\right)$, $P_{\min}$ is the smallest error probability when the synthesized channel is carrying *i* bits. The encoder of bit-level-construction-based non-binary polar codes is the same as for symbol-level construction. At the decoder, Algorithm 4 is also used to search for a valid decoding symbol when decoding the synthesized channel carrying *i* bits.
**Algorithm 1** Degrading procedure of synthesized channels**Input:** DMC *W*, a bound of output size μ, code length $N=2^{n}$, channel index *j* with binary representation ${\langle b_{j,1},b_{j,2},\ldots ,b_{j,n}\rangle }_{2}$.**Output:** A DMC $Q_{N}^{\left(j\right)}$ that is degraded with respect to the synthesized channel $W_{N}^{\left(j\right)}$.  1:  Q←degrade(W,μ)  2:  **for** i=1:n **do**  3:      **if** $b_{j,i}=0$ **then**  4:         $W\leftarrow Q^{-}$  5:      **else**  6:         $W\leftarrow Q^{+}$  7:      **end if**  8:      Q←degrade(W,μ)  9:  **end for**10:  **return** *Q*

**Algorithm 2** The degrade function**Input:** DMC W:X→Y, where |Y|=L, a bound of output size μ.**Output:** A degrade channel $Q:X\to Y^{\prime }$, where $|Y^{\prime }|\leq \mu$.
**for** i=1:L **do**2:   **for** $j=0:2^{q}-1$ **do**
     $d\left[i\right]\left[j\right]\leftarrow W\left(y_{i}|j\right)$4:   **end for**

**end for**
6:

$Y^{\prime }\leftarrow Y$


**while** $|Y^{\prime }|>\mu$ **do**8:   $\left\{y_{1},y_{2}\right\}\leftarrow \mathrm{GetOutputwithMinDeltaC}\left(Q\right)$
   **for** $j=0:2^{q}-1$ **do**10:     $d\left[y_{\mathrm{merge}}\right]\left[j\right]=d\left[y_{1}\right]\left[j\right]+d\left[y_{2}\right]\left[j\right]$
   **end for**12:   $Y^{\prime }\leftarrow y_{\mathrm{merge}}$
   $Y^{\prime }\leftarrow Y^{\prime }\setminus \left\{y_{1},y_{2}\right\}$14:
**end while**

Construct *Q* according to the probabilities.16:**return** *Q*

*Example:* Given an N=8,K=4, MR-based non-binary polar code over GF(8) and the quantization level μ=8. Table 1 lists the transition probabilities of the 6-th synthesized channel $Q_{8}^{6}\left(y|v^{\prime }\right)$. Assuming that $C(Q_{8}^{6})=2$ bits, there are three patterns for the 6-th channel to carry two information bits, (1,1,0),(1,0,1), and (0,1,1). When $T^{\left(6\right)}=\left(1,1,0\right)$, $v^{\prime }\in \left\{0,1,2,3\right\}$, according to Equations (22) and (23), we can calculate that $P_{\left(1,1,0\right)}=0.134275$. Similarly, when $T^{\left(6\right)}=\left(1,0,1\right)$, $v^{\prime }\in \left\{0,1,4,5\right\}$, $P_{\left(1,0,1\right)}=0.1346$, and when $T^{\left(6\right)}=\left\{0,1,1\right\}$, $v^{\prime }\in \left\{4,5,6,7\right\}$, $P_{\left(0,1,1\right)}=0.13455$. Therefore, the 18-th and 19-th bits are selected as information bits.

**Figure 3:**
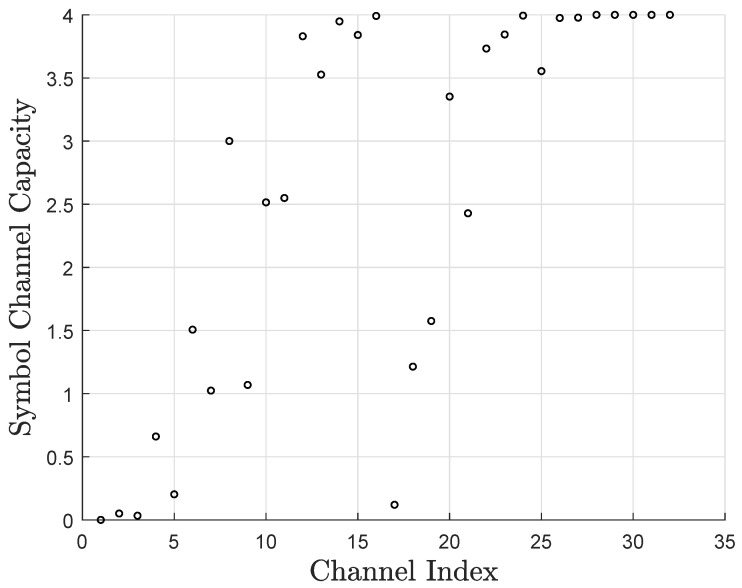
Synthesized channel capacities of non-binary polar codes over the AWGN channel.

Figure 3 shows the synthesized channel capacities of 16-ary polar codes over the AWGN channel with binary phase shift keying (BPSK) modulation, where N=32, the signal-to-noise ratio (SNR) is 3.0 dB, and μ=32. Since the sum capacities of each synthesized channel in Figure 2 and Figure 3 are equal, we can directly compare the differences in their symbol channel capacities. Obviously, the two approaches produce different results. The capacity calculation under the erasure channel is simple but not precise. On the contrary, the CD construction is more complex but yields more accurate results.
**Algorithm 3** Obtain information bit set of *j*-th synthesized channel**Input:** The transition probability $Q_{N}^{\left(j\right)}\left(y|v\right)$ of *j*-th synthesized channel, where y∈Y,|Y|=μ,v∈ GF$\left(2^{q}\right)$, *j*-th synthesized channel carried information bits *i*.**Output:** $T^{\left(j\right)}$.
$P_{T}\leftarrow 0$
$P_{max}\leftarrow 0$3:$P_{min}\leftarrow 1$
**for** $k=0:2^{q}-1$ **do**
   **if** $\sum_{m=1}^{q}b_{k,m}=i$ **then**6:     $P_{T}\leftarrow 0$
     **for** $y=y_{1}:y_{\mu }$ **do**
        $P_{\max}\leftarrow 0$9:        **for** $v=0:2^{q}-1$ **do**
          **if** IsVlegal(v,k)=1 **then**
             **if** $Q_{N}^{\left(j\right)}\left(y|v\right)>P_{\max}$ **then**12:               $P_{T}\leftarrow P_{T}+P_{\max}$
               $P_{\max}=Q_{N}^{\left(j\right)}\left(y|v\right)$
             **else**15:               $P_{T}\leftarrow P_{T}+P_{\max}$
             **end if**
          **end if**18:        **end for**
     **end for**
     $P_{T}\leftarrow P_{T}/2^{i}$21:     **if** $P_{T}<P_{\min}$ **then**
        $P_{\min}\leftarrow P_{T}$
        **for** m=1:q **do**24:          $T_{m}^{\left(j\right)}\leftarrow b_{k,m}$
        **end for**
     **end if**27:   **end if**
**end for**
**return** $T^{\left(j\right)}$
**Algorithm 4** IsVlegal**Input:** v,k.**Output:** result.
result←1
**for** m=1:q **do**
   **if** $b_{k,m}=0$ **then**4:     **if** $b_{v,m}\neq 0$ **then**
        result←0
        break
     **end if**8:   **end if**
**end for**
**return** result

### 3.2. Error-Probability-Based Bit-Level Construction

Although the CD construction achieves high accuracy with a large quantization level μ, it also leads to a high complexity. To reduce the complexity of the construction algorithm, we consider an error-probability-based bit-level construction for MC construction. Meanwhile, we make all the synthesized channels carry information bits with the same pattern. For example, if the *j*-th synthesized channel carries 0<i<q bits, its input symbol $v_{j}\in \left\{0,1,\ldots ,2^{i}-1\right\}$.

Consider an (N,K,A) non-binary polar code over GF($2^{q}$). The set of block error events under SC decoding is defined as

(24) $$E≜\left\{\left(v_{1}^{N},y_{1}^{N}\right):{\hat{v}}_{A}\left(v_{1}^{N},y_{1}^{N}\right)\neq v_{A}\right\}.$$

Let $P_{e}\left(N,K,A\right)=P\left(E\right)$ denote the BLER of the (N,K,A) polar code. The set of block error events can be enlarged as $E\subset {⋃}_{j\in A}E_{j}$, where the single-symbol error event $E_{j}$ is defined as

(25) $$\begin{array}{cc} E_{j}≜ & \left\{\left(v_{1}^{N},y_{1}^{N}\right):W_{N}^{\left(j\right)}\left(y_{1}^{N},v_{1}^{j-1}|v_{j}\right)\right.\\ \; & \left.\leq W_{N}^{\left(j\right)}\left(y_{1}^{N},v_{1}^{j-1}|v_{j^{\prime }}\right),v_{j^{\prime }}\neq v_{j}\right\}. \end{array}$$

Let $P_{e,i}\left(j\right)$ denote the symbol error probability when the *j*-th synthesized channel carries *i* bits. Obviously, $P_{e,i_{1}}\left(j\right)\leq P_{e,i_{2}}\left(j\right)$ for any $i_{1}<i_{2}$ and 0≤j<N. $P_{e,i}\left(j\right)$ can be easily obtained by the MC method and the CD method. For the CD method, a DMC is obtained from the synthesized channel $Q_{N}^{\left(j\right)}$, and $P_{e,i}\left(j\right)$ can be calculated by

(26) $$P_{e,i}\left(j\right)=\frac{1}{2^{i}}\sum_{y\in Y}\sum_{\begin{array}{c} v=0,\\ v\neq v_{\max} \end{array}}^{2^{i}-1}Q_{N}^{\left(j\right)}\left(y|v\right),$$

where

(27) $$v_{\max}=\underset{v\in \{0,1,\ldots 2^{i}-1\}}{\arg\max}\;Q_{N}^{\left(j\right)}\left(y|v\right).$$

The BLER for bit-level construction under the SC decoding can be upper bounded as

(28) $$P_{e}\left(N,K,A_{1}\right)=P\left(E\right)\leq \sum_{j\in A_{1}}P\left(E_{j}\right)\approx \sum_{j\in A_{1}}P_{e,I(j)}\left(j\right),$$

subject to (14).

Our scheme aims to obtain an optimal information symbol set $A_{1}$ and number of carried bits I(j) to minimize the upper bound of the total probability $P_{e}$. To address this problem, we compute a probability $P_{b}\left(s\right)$, which represents the contribution to the upper bound of the error probability when selecting the *s*-th bit, namely $A_{1}$.

(29) $$P_{b}\left(s\right)=\frac{P_{e,i+1}\left(j\right)-P_{e,i}\left(j\right)}{1-P_{e,i}\left(j\right)}$$

where s=(j−1)q+i+1, 1≤s≤Nq, and $P_{e,0}\left(j\right)=0$. In the case of 0<i<q, $P_{b}\left(s\right)$ is a conditional probability $P({\hat{u}}_{s}\neq u_{s}|{\hat{u}}_{s-1}=u_{s-1},\ldots ,{\hat{u}}_{s-i}=u_{s-i})$ when the *j*-th synthesized channel carries i+1 bits. Consequently, the BLER under SC decoding can be further upper bounded as

(30) $$P\left(E\right)\leq \sum_{j\in A_{1}}P_{e,I(j)}\left(j\right)=\sum_{j\in A_{1}}\prod_{s\in B}P_{b}\left(s\right)=P_{UB},$$

where B is the information bit set and |B|=Kq, and $P_{UB}$ is the BLER upper bound under the SC decoding. We can use the upper bound of the error probability as a metric to generate $A_{1}$ and B by choosing Kq bit indexes with the smallest $P_{b}\left(s\right)$. Note that for some $s_{1}>s_{2},s_{1}=\left(j-1\right)q+i_{1}+1,s_{2}=\left(j-1\right)q+i_{2}+1,0\leq i_{2}<i_{1}<q$, there are $P_{b}\left(s_{1}\right)\leq P_{T}<P_{b}\left(s_{2}\right)$, where $P_{T}$ is the Kq-th smallest probability. This means that $s_{1}\in B,s_{2}\notin B$, which means the construction result does not satisfy the fixed carry pattern. Therefore, we must ensure that, for this case, $s_{2}$ is selected earlier than $s_{1}$ when we choose the Kq smallest $P_{b}\left(s\right)$, which is different from the construction scheme in [12,14]. The overall procedure of the error-probability-based bit-level construction algorithm is shown in Algorithm 5.

**Figure 4:**
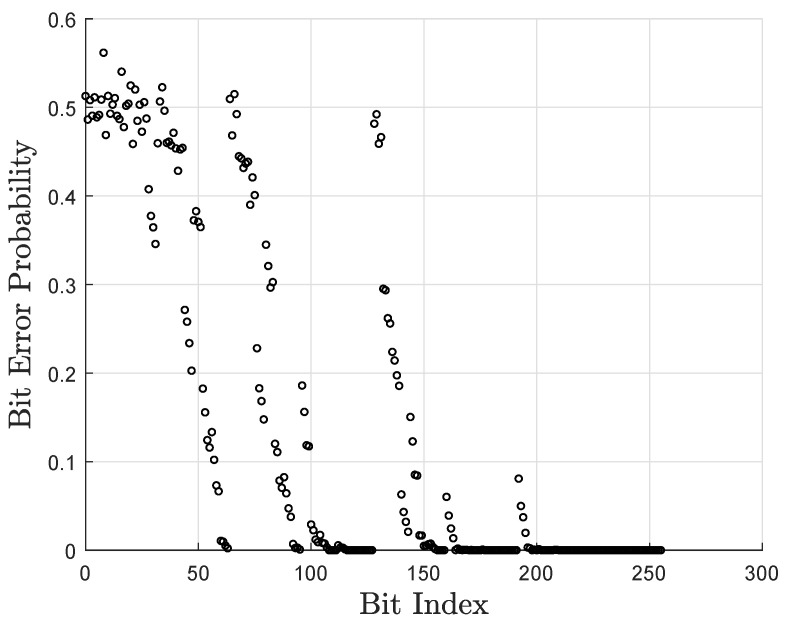
The error probability of each decoding bit under the SC decoder.

Figure 4 shows the error probability of each decoding bit under the SC decoder, where N=64 symbols, K=32 symbols over GF(16), and the $E_{b}/N_{0}=2.0$ dB.
**Algorithm 5** Error-Probability-Based Bit-Level Construction for Non-binary Polar Codes**Input:** $N,K,q,\{P_{e,i}\left(j\right):1\leq i\leq q,1\leq j\leq N\}$.**Output:** $A_{1},B,I$.
$A_{1}\leftarrow \emptyset ,B\leftarrow \emptyset$
**for** s=1:Nq **do**
   Calculate $P_{b}\left(s\right)$ according to (29).
**end for**5:Generate B by selecting the Kq indices *s* with the smallest $P_{b}\left(s\right)$ values.
**for** j=1:N **do**
   I(j)=0
   **for** i=0:q−1 **do**
     **if** ((j−1)q+i+1)∈B **then**10:        I(j)=I(j)+1
     **end if**
   **end for**
   **if** I(j)≠0 **then**     $A_{1}\leftarrow j$15:   **end if**
**end for**
B←∅
**for** j=1:N **do**
   **for** i=0:q−1 **do**20:     **if** $j\in A_{1}\;\&\;\left(i+1\right)\leq I\left(j\right)$ **then**
        B←((j−1)q+i+1)
     **end if**
   **end for**
**end for**

## 4. Construction for MR-Based Non-Binary Polar Codes with High-Order Modulation and Probabilistic Shaping

To achieve high spectral efficiency, MR-based non-binary polar codes combined with high-order modulation are considered. It is well known that the optimal input distribution for AWGN channels is Gaussian. As the spectral efficiency increases, the performance loss caused by the use of uniform signaling can be up to 1.53 dB [23,24]. Probabilistic shaping is a technique developed to mitigate this loss. In this section, we introduce the code construction for MR-based non-binary polar codes with BICM and probabilistic shaping.

### 4.1. Hybrid MLC and BICM Coded Modulation Framework

**Figure 5:**
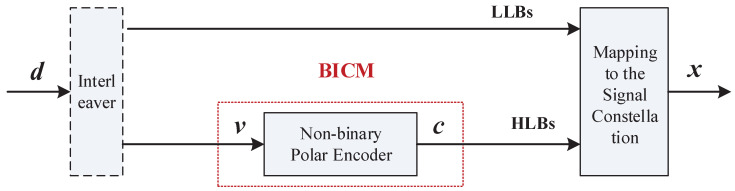
System model of shaping non-binary polar codes with hybrid MLC and BICM scheme.

Yuan et al. proposed a hybrid MLC and BICM coded modulation framework based on Ungerboeck set partitioning, to meet requirements for higher spectral efficiency and higher throughput in future 6G communications in [28]. Figure 5 shows the hybrid MLC-BICM coded modulation system model combined with non-binary polar codes. MLC incorporates Ungerboeck set partitioning, which divides the signal constellation X into $2^{q-1}$ subsets through q−1 partitioning steps. Let $d_{j},\left(j=0,1,\ldots ,q-1\right)$ denote the minimum square Euclidean distances of the *j*-th level subsets, where the average energy of the original constellation is normalized to 1. The *j*-th level bit error rate (BER) of the intra-subset can be calculated by

(31) $$P\left(d_{j}\right)\approx \frac{1}{2}\mathrm{erfc}\left(\sqrt{\frac{E_{s}d_{j}}{4N_{0}}}\right)=\frac{1}{2}\mathrm{erfc}\left(\sqrt{\frac{\rho E_{b}d_{j}}{4N_{0}}}\right),$$

where $E_{s}$ is the average energy of the constellation X, $E_{b}$ is the average energy of each information bit [30], and ρ is the corresponding spectral efficiency (SE) in bits/two-dimensional (2D) symbol. Therefore, at a certain level of the subset partitioning chain, the signals within the subset can be detected by a hard decision with high reliability, because the BER is reliable enough, such as a BER $\leq 10^{-5}$. We divide the index bits of the transmitted signal into two groups: the high-level bits (HLBs) used to index the subset, and the low-level bits (LLBs) used to index the signal in the subset. To achieve better performance, we apply Gray labeling for both LLBs and HLBs.

### 4.2. Probabilistic Shaping for MR-Based Non-Binary Polar Codes

**Figure 6:**
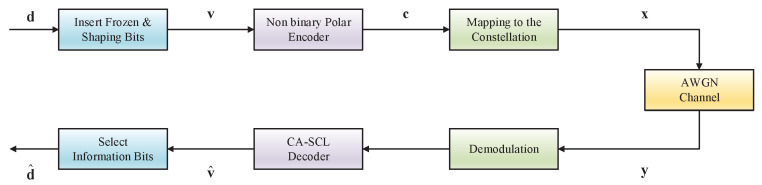
System model of shaping non-binary polar codes with BICM scheme.

Figure 6 depicts a block diagram of shaping non-binary polar codes with the BICM scheme. Suppose that a two-dimensional signal constellation X of size $\left|X\right|=2^{q}$ is used. In other words, the field matches the modulation order. In order to ensure that the transmitted symbols follow a carefully optimized distribution, such as the Maxwell–Boltzmann distribution

(32) $$P_{X}\left(x\right)=\frac{\exp\left(-{\lambda ∥x∥}^{2}\right)}{\sum_{\tilde{x}\in X}\exp\left(-\lambda ∥\tilde{x}{∥}^{2}\right)},$$

where λ≥0, the information sequence $d=d_{1},d_{2},\ldots ,d_{K}$ needs to contain some shaping symbols. After inserting the shaping symbols and frozen symbols, the input sequence $v=v_{1},v_{2},\ldots ,v_{N}$ can be obtained. The codewords are generated by $c_{1}^{N}=v_{1}^{N}G_{N}$. A signal mapper π maps the polar coded symbols $c_{i},1\leq i\leq N$ to modulated symbols $x_{i}=\pi \left(c_{i}\right)\in X$. We define a symbol set S,A∪F∪S={1,2,…,N}. By reformulating (8) as $v_{1}^{N}G_{N}+\pi^{-1}\left(x_{1}^{N}\right)=0$, we can regard this as a polar codeword $c_{1}^{N}$ transmitted over a $2^{q}$-ary symmetric channel with transition probability $P_{X}\left(x\right)$, where $\pi^{-1}\left(x_{1}^{N}\right)$ is the noise sequence and the channel output is the all-zero vector. Therefore, we can use symbol-level or bit-level construction to obtain the shaping set S and use the SC decoder to obtain the shaping symbol value. Note that $\left|S|=\lceil N\left(1-H_{2^{q}}\left(P_{X}\left(x\right)\right)\right)\right\rceil$ symbols, where $H\left(P_{X}\left(x\right)\right)=\sum_{x\in X}-P_{X}\left(x\right)\log_{2^{q}}\left(P_{X}\left(x\right)\right)$. At the decoder, the initial probabilities are generated based on the channel output $y_{1}^{N}$ as

(33) $$W\left(x_{i}|y_{i}\right)\propto W_{\mathrm{AWGN}}\left(y_{i}|x_{i}\right)P_{X}\left(x_{i}\right),$$

where $W_{\mathrm{AWGN}}\left(y_{i}|x_{i}\right)$ is the transition probability over the AWGN channel.

For a hybrid MLC and BICM coded modulation framework, since the LLBs are without channel coding, probabilistic shaping can only be achieved at the HLBs. Based on the results of (32), we can calculate the probability of each subset. However, the probability distribution of each subset is close to a uniform distribution.

### 4.3. Code Design

**Figure 7:**
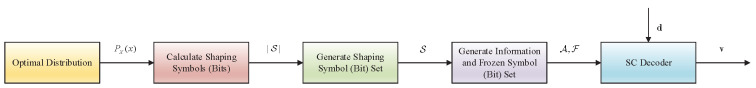
The code design procedure of MR-based non-binary polar codes with high-order modulation and probabilistic shaping.

In this part, we introduce the code design of the MR-based non-binary polar codes with high-order modulation and probabilistic shaping in detail, as illustrated in Figure 7.

For the symbol-level shaping method, we first calculate the number of shaping symbols |S| according to the optimal distribution $P_{X}\left(x\right)$. Then, we use the symbol-level construction over the $2^{q}$-ary symmetric channel to obtain the shaping symbol set S. Note that both methods mentioned above can be used. Then, for the AWGN channel, we calculate the capacities or error probabilities for each synthesized channel and assign the best synthesized channels (excluding the ones in S) to A. The remaining synthesized channels are assigned to F. Once the symbol sets S,A,F are known, we can use the SC decoder to calculate the values of the shaping symbols. For this purpose, we regard $v_{j},j\in A\cup F$ as frozen symbols and $P_{X}\left(x\right)$ as the channel observation of an all-zero vector.

For the bit-level shaping method, we define a shaping bit set $S_{\mathrm{bit}}$ and a frozen bit set $F_{\mathrm{bit}}$, $S_{\mathrm{bit}}\cup B\cup F_{\mathrm{bit}}=\left\{1,2,\ldots ,Nq\right\}$. The number of shaping bits can be calculated by $|S_{\mathrm{bit}}|=\left\lceil N\left(q-H_{2}\left(P_{X}\left(x\right)\right)\right)\right\rceil$. The shaping bit set $S_{\mathrm{bit}}$, the information bit set B, and the frozen bit set $F_{\mathrm{bit}}$ can be obtained through the bit-level construction. Due to the bit-level construction, a synthetic channel may contain both information bits and shaping bits simultaneously, which is the main difference compared with the symbol-level shaping method.

## 5. Simulation Results

In this section, the performance of the proposed construction scheme based on the CD method and MC method for MR-based non-binary polar codes is evaluated. Assume that binary phase shift keying (BPSK) modulation is used over the AWGN channel. For comparison, the performance of the original Monte-Carlo construction [8], binary polar codes, and the construction scheme in [12] are also provided. Note that the binary polar codes were constructed based on the 5G construction [4]. The design SNR of all constructions was 2.0 dB, the CRC length and the list size were both set to 8. At the decoder, the symbol-level decoding algorithm was used for the non-binary polar codes. Therefore, the decoding complexity of the proposed construction scheme was the same as that of the symbol-level construction scheme.

**Figure 8:**
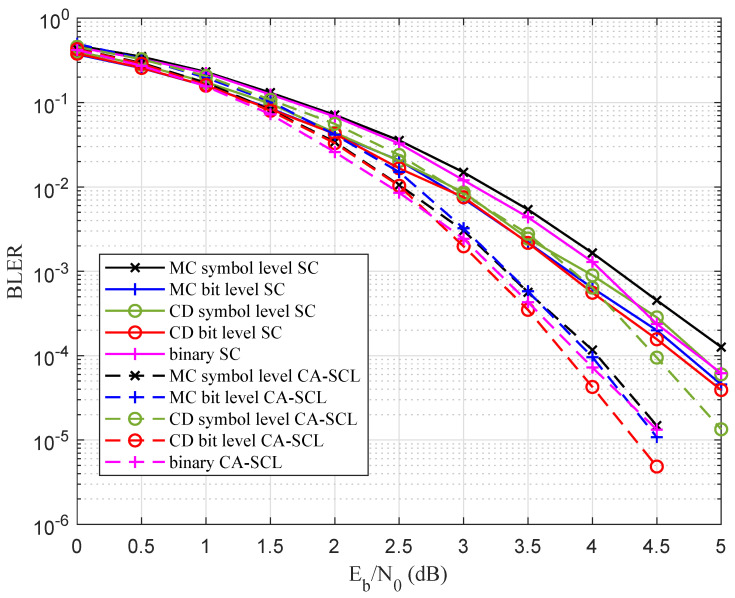
Performance comparison among the bit-level construction and symbol-level construction.

Figure 8 depicts the performance comparison between the symbol-level construction and the bit-level construction when N=32 and R=1/4 over GF(16). The SC decoding and CA-SCL decoding algorithms were used. When using the SC decoder, the bit-level MC construction and the bit-level CD construction proposed in this work exhibited the same performance, which was 0.2 dB better than the symbol-level MC construction. When using the CA-SCL decoder, the bit-level CD construction also achieved the best BLER performance, outperforming the MC method by 0.2 dB. Since the quantization level μ=32, the performance loss of the CD scheme increased with the code length and rate.

**Figure 9:**
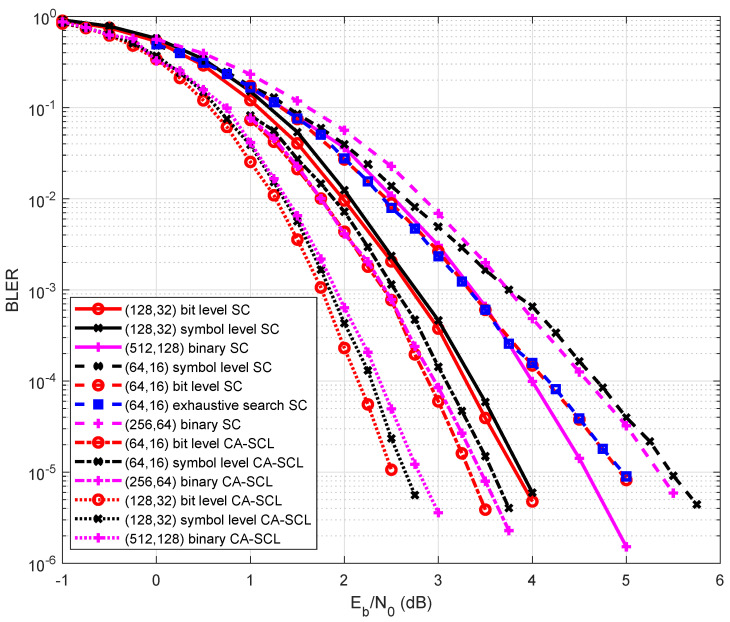
Performance comparison among the proposed scheme, conventional scheme, and binary polar codes, where N=128,64, R=1/4 over GF(16), and the SC and CA-SCL decoding algorithms were used.

With regard to Figure 9, when N=64,R=1/4 over GF(16), and the SC decoding algorithm was used, the symbol-level construction scheme had a similar BLER performance compared with the binary polar code, and the bit-level construction scheme performed 0.5 dB better than the above two schemes at BLER $=1\times 10^{-5}$. The proposed scheme and the exhaustive search scheme had the same BLER performance, but the proposed scheme had a lower complexity. When N=128, the proposed bit-level construction scheme and symbol-level construction scheme had the same BLER performance, which was 0.75 dB better than that of the binary polar codes.

Figure 9 also shows the performance comparison using the CA-SCL decoding algorithm. Since the CRC bits occupied the information sub-channels, the error probability upper bound of the proposed construction scheme increased. When N=64, the bit-level construction scheme achieved a performance gain of 0.25 dB and 0.15 dB compared with symbol-level construction and binary polar codes. When N=128, the bit-level construction scheme also achieved a gain of 0.15 dB compared with the symbol-level construction scheme, which was 0.25 dB better than the binary polar code in the high SNR region.

**Figure 10:**
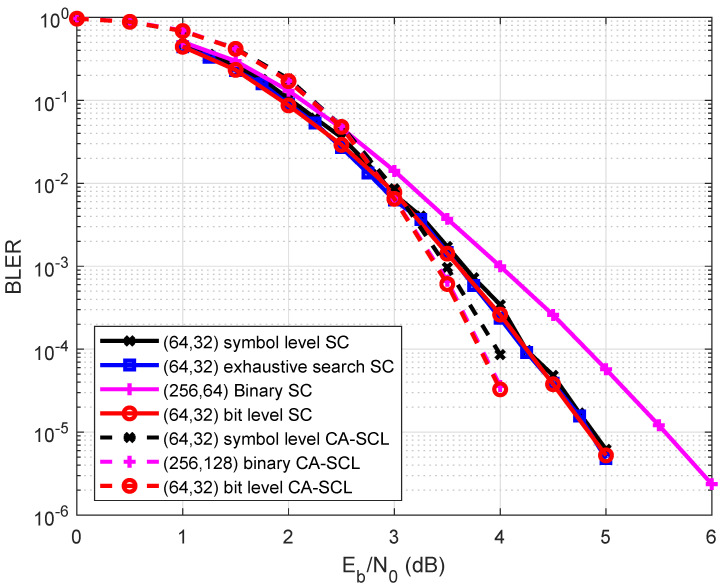
Performance comparison among the proposed scheme, conventional scheme, and binary polar codes, where N=64, R=1/2 over GF(16), and the SC and CA-SCL decoding algorithms were used.

In Figure 10, when N=64,R=1/2, with the symbol-level construction scheme, the bit-level construction and the exhaustive search scheme had the same BLER performance under the SC decoding algorithm, which was 0.7 dB better than the binary polar code. Using the CA-SCL decoding with the CRC length of 24, the proposed scheme had the same BLER performance as the binary polar code, and outperformed the symbol-level construction by 0.2 dB at BLER = $10^{-4}$.

**Figure 11:**
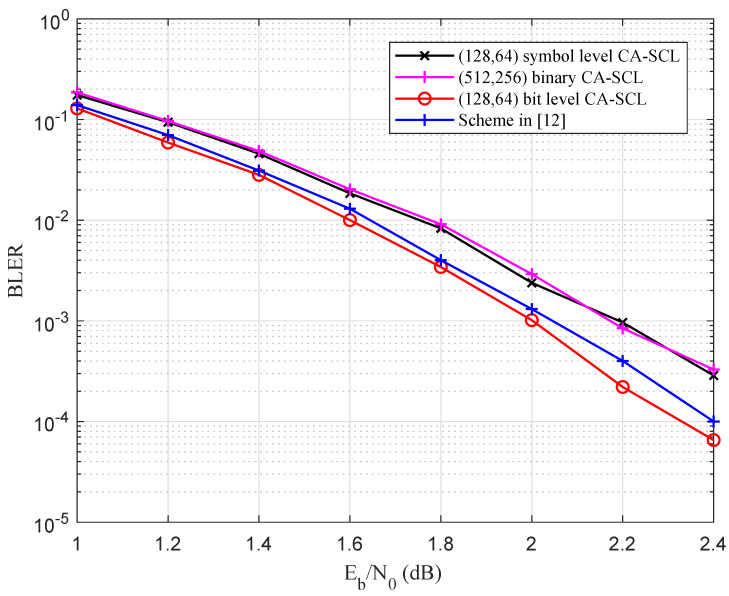
Performance comparison among the proposed scheme, conventional scheme, and binary polar codes, and scheme in [12] where N=128, R=1/2 over GF(16) and the CA-SCL decoding algorithm was used.

Figure 11 shows the performance comparison between the proposed construction scheme and the scheme in [12], where N=128,R=1/2, and the list size was 16. The proposed scheme was 0.1 dB better than the scheme in [12] and 0.2 dB better than the symbol-level construction scheme and the binary polar code at BLER $=2\times 10^{-4}$. Since the input symbols in our scheme satisfy $v_{j}\in \left\{0,1,...,2^{i}-1\right\}$, the decoding search space was reduced compared with that in [12].

**Figure 12:**
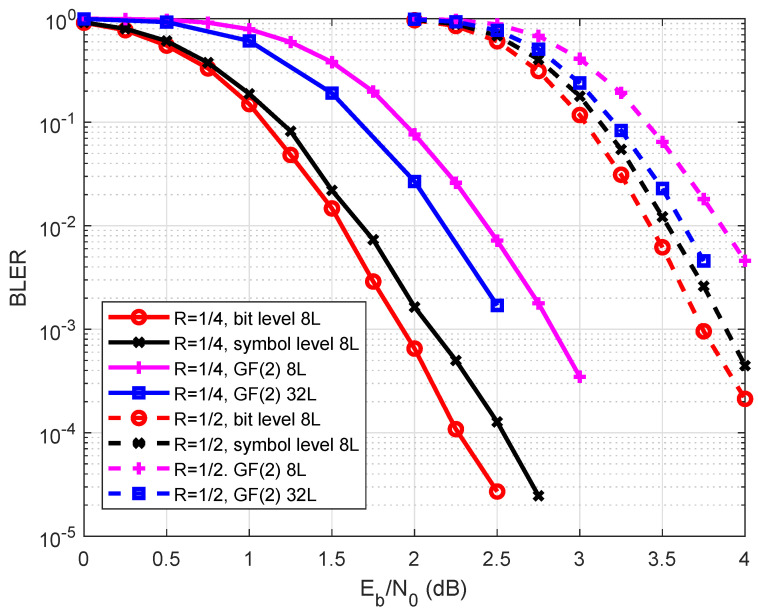
Performance with 16-QAM over the AWGN channel.

We now evaluate the code performance under 16-QAM modulation. Two codes of length N=512 over GF(16) with code rates 1/4 and 1/2, respectively, were evaluated. Assume that the CA-SCL decoding algorithm was used. The performance of both non-binary polar codes and binary polar codes with bit interleaved coded modulation (BICM) is shown in Figure 12. When R=1/4, the proposed scheme achieved performance gains of 0.25 and 1 dB compared with the symbol-level construction and binary polar codes. When R=1/2, our scheme had performance gains of 0.2 dB and 0.5 dB, respectively.

**Figure 13:**
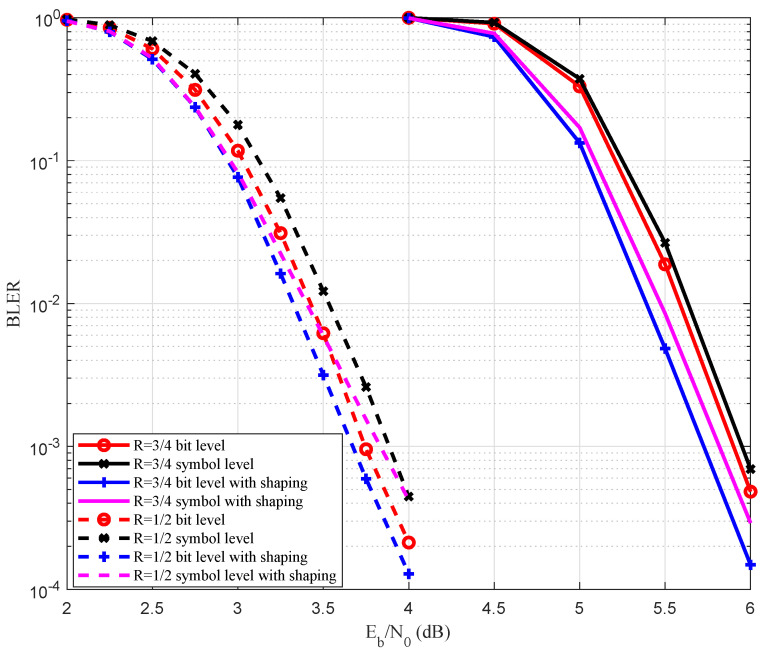
Probabilistic shaping performance with 16-QAM over the AWGN channel.

The performance of the probabilistic shaped MR non-binary polar codes is shown in Figure 13. We compared the shaping gain and coding gain of the bit-level construction and the symbol-level construction schemes. When N=512, R=1/2, both bit-level construction and symbol construction had 0.1 dB shaping gain at BLER $=1\times 10^{-3}$, while the coding gain was 0.2 dB. When N=512, R=3/4, both the bit-level construction and symbol-level construction had a 0.25 dB shaping gain at BLER $=1\times 10^{-3}$, while the coding gain was 0.1 dB. In a word, the bit-level construction had a significant shaping gain with a high coding rate.

**Figure 14:**
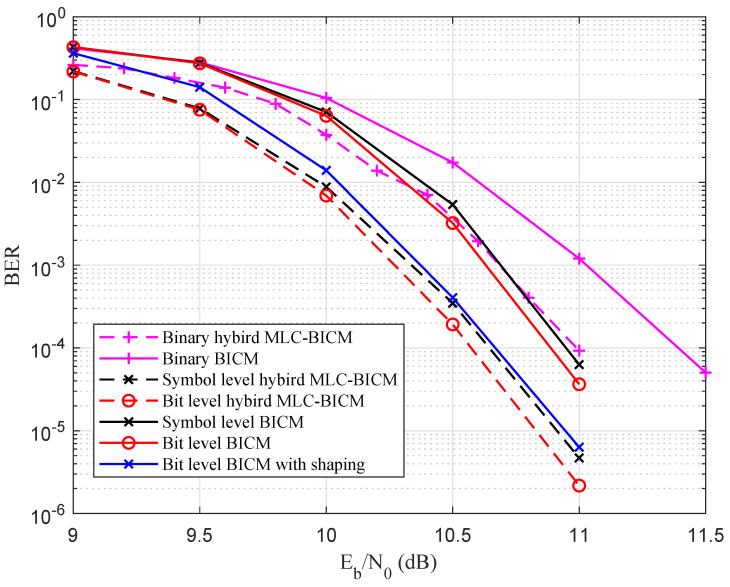
Performance comparison between the BICM and hybrid MLC-BICM with 64-QAM over the AWGN channel.

A coded modulation performance comparison between BICM and hybrid MLC-BICM with 64-QAM modulation is shown in Figure 14. For the BICM system, the code length N=256,R=5/6, the crc length was 12, and the list size was 8. For the hybrid MLC and BICM system, the signal constellation was partitioned into subsets of size 4, which meant that 512 out of 1280 information bits were uncoded and the rest of the information bits were protected using a non-binary (binary) polar code with a rate of 3/4. The simulation results show that the hybrid MLC-BICM scheme was about 0.4 dB better than the BICM scheme at a bit error rate (BER) $=1\times 10^{-4}$. Meanwhile, for the BICM system, the bit-level construction achieved a 0.25 dB gain at BER $=1\times 10^{-4}$.

## 6. Conclusions

This paper presents an improved bit-level construction for MR-based non-binary polar codes, incorporating both CD and MC methods. The number of transmitted bits is determined by evaluating the error probability of each synthesized channel and the lower bound of its capacity, thereby minimizing the symbol error probability. For practical implementation, MC-based bit-level construction is additionally proposed to reduce computational complexity. The simulation results showed that the proposed construction scheme outperformed binary polar codes and conventional non-binary polar codes with BPSK under both SC and CA-SCL decoding algorithms. Furthermore, we incorporate probabilistic shaping within our bit-level construction framework to address shaping loss in high-order modulation systems. The simulation results demonstrated that the proposed architecture achieved substantial shaping gains for high coding rates, while maintaining notable coding gains at lower coding rates. 

## Figures and Tables

**Table 1:** The transition probabilities of the 6-th synthesized channel $W(y|v^{\prime })$.

$Q_{8}^{6}\left(y|v^{\prime }\right)$	$v^{\prime }=0$	$v^{\prime }=1$	$v^{\prime }=2$	$v^{\prime }=3$	$v^{\prime }=4$	$v^{\prime }=5$	$v^{\prime }=6$	$v^{\prime }=7$
$y=y_{1}$	0.8357	0.0269	0.0297	0.0307	0.0298	0.0154	0.0141	0.0177
$y=y_{2}$	0.03	0.0294	0.027	0.8347	0.02	0.0139	0.015	0.03
$y=y_{3}$	0.03	0.0309	0.8362	0.026	0.014	0.0179	0.0298	0.0152
$y=y_{4}$	0.018	0.0139	0.0156	0.0303	0.0299	0.0302	0.0266	0.8355
$y=y_{5}$	0.014	0.018	0.03	0.015	0.029	0.03	0.837	0.027
$y=y_{6}$	0.016	0.0302	0.018	0.014	0.0269	0.8349	0.03	0.03
$y=y_{7}$	0.026	0.8355	0.03	0.031	0.016	0.03	0.0175	0.014
$y=y_{8}$	0.0303	0.0152	0.0135	0.0183	0.8344	0.0277	0.03	0.0306

## Data Availability

The data are contained within the article.

## Abbreviations

The following abbreviations are used in this manuscript:

MR	Multiplicative Repetition
BI-DMC	Binary-input discrete memoryless channels
SC	Successive cancellation
CRC	Cyclic redundancy check
CA-SCL	Cyclic redundancy check-aided successive cancellation list
LDPC	Low-density parity check
5G	fifth generation
RS	Reed–Solomon
AWGN	Additive White Gaussian Noise
MC	Monte-Carlo
GA	Gaussian approximation
PW	Polarized weight
CD	Channel degradation
BLER	Block error rate
MLC	Multilevel coding
BICM	Bit-interleaved coded modulation
BPSK	Binary phase-shift keying
BER	Bit error rate
